# Relationship between a Prolonged Corrected QT Interval and Mortality in Patients Presenting with Syncope at the Emergency Department

**DOI:** 10.1155/2021/5441670

**Published:** 2021-11-24

**Authors:** Pınar Yeşim Akyol, Hüseyin Acar, Adem Çakır, Yusuf Şahin, Zeynep Karakaya, Fatih Esad Topal

**Affiliations:** ^1^Department of Emergency Medicine, Izmir Katip Çelebi University Atatürk Training and Research Hospital, İzmir, Turkey; ^2^Department of Emergency Medicine, Republic of Turkey, The Ministry of Health Basaksehir Cam and Sakura City Hospital, Istanbul, Turkey; ^3^Department of Emergency Medicine, The Ministry of Health Sanlıurfa Training and Research Hospital, Sanlıurfa, Turkey

## Abstract

**Background:**

Syncope is a common symptom in emergency department patients. Among various etiological factors, cardiac causes have the highest risk of mortality. The corrected QT interval is considered an independent predictor of mortality for many diseases.

**Objectives:**

Analyze QT interval analysis of patients presenting to the emergency department with syncope.

**Methods:**

In this prospective observational study, patients who presented to the emergency department with syncope between January 1, 2018, and January 1, 2019 were included.

**Results:**

The median age was 64 (49-78) years, and 58.8% of patients were male. The corrected QT interval (QTc) in patients with coronary artery disease and chronic obstructive pulmonary disease was longer than those without. There was no statistically significant association between hypertension, diabetes, stroke, thyroid disease, and prolonged QTc. Patients who did not survive had significantly prolonged QT intervals. According to ROC analysis, sensitivity of >440.5 ms QTc values in predicting mortality was 86% and specificity was 71% (AUC = 0.815; 95%CI = 0.71 − 0.91; *p* < 0.001).

**Conclusions:**

Patients admitted to emergency department with syncope and a prolonged QTc are associated with a higher mortality rate and thus can provide us with an important guide for the management of these patients.

## 1. Introduction

Syncope is a common symptom among patients admitted to emergency department (ED) [1]. Among various etiologies, cardiogenic syncope has the highest mortality rate [2] . Although many studies have examined the relationship between QRS morphology and syncope, studies on corrected QTc in syncope have been limited [3]. Although QTc is considered an independent indicator of mortality for many diseases such as coronary artery disease (CAD), diabetes mellitus (DM), and congestive heart failure (CHF), its effect on mortality in syncope patients is unclear [4–6].

The QT interval, which is an indicator of ventricular repolarization time, is defined as the distance between the beginning of QRS and the end of the T wave in ECG. Because of the strong influence of the heart on the QT interval, a corrected QT interval, adjusted for heart rate, is commonly used. QT prolongation can lead to the development of Torsades de Pointes, a rare polymorphic ventricular tachycardia [7]. Congenital or acquired causes are included in the etiology of QT interval prolongation, also known as long QT syndrome (LQTS) [8]. Electrolyte abnormalities, sinus node dysfunction, atrioventricular blocks, myocardial ischemia, intoxications, and medication use are among the acquired etiological causes [9, 10].

This study is aimed at investigating the Relatıonship between QTc and mortality in patients who have unpredicted QT prolongation and can be overlooked admitted to the ED with syncope.

## 2. Methods

### 2.1. Study Setting

This prospective study was carried out in the ED of Izmir Katip Çelebi University Atatürk Training and Research Hospital between January 1, 2018, and January 1, 2019.

### 2.2. Study Population

#### 2.2.1. Inclusion Criteria

Patients aged 18 years and over who presented to the ED with a complaint of syncope were included in this study after the written consent was obtained. Syncope was defined as a transient, self-limited loss of consciousness with an inability to maintain a postural tone that is followed by spontaneous recovery [11].

#### 2.2.2. Exclusion Criteria

Patients under 18 years of age, pregnant women, patients with a medical history of the long or short QT interval, patients with a history of medication use that may affect QT, and patients with unknown medical history were excluded from the study.

### 2.3. Data Collection

ECG was taken in the first 10 minutes of the patients' admission to the ED and evaluated by the emergency physician. QTc is calculated by computer using Bazett's formula: (QTc = QT/√RR) [12].

Blood pressure, pulse rate, and oxygen saturation of all patients were recorded in the study form.

### 2.4. Statistical Analysis

The data were analyzed in the SPSS 23.0 for Windows® statistics program (IBM Inc., Chicago, IL, USA). Number, percentage, median, minimum, and maximum were used in the presentation of descriptive data. Pearson chi-square tests and Fisher's exact tests were used to compare categorical data. Mann–Whitney *U* tests were used to compare numerical data. ROC analysis was used to describe the relationship between mortality and QTc values in syncope patients. Results were considered significant at *p* < 0.05.

### 2.5. Ethical Considerations

Ethics committee approval was obtained from the Izmir Kâtip Celebi-Non-Interventional Clinical Studies Institutional Review Board (Ethics committee number: 2016-GOKAE-0141). All procedures performed in this study were in accordance with the ethical standards of the institutional and/or national research committee and with the 1964 Helsinki Declaration and its later amendments.

## 3. Results

A total of 165 patients are included in this study; 58.8% (*n* = 97) are males and 41.2% (*n* = 68) are females. The median age of the patients was 64 (IQR: 49-78; range: 18-93 years). No statistically significant difference was found between men and women in terms of QTc. The vital signs and demographic characteristics of the patients at the time of presentation are given in Table 1. Age distribution was similar in the surviving and nonsurviving patient groups. Systolic blood pressure and diastolic blood pressure, however, were significantly reduced, and pulse rate significantly elevated in patients that did not survive (*p* = 0.042, *p* = 0.004, and *p* = 0.015, respectively) (Table 1).

Median systolic blood pressure was 119 (IQR: 102-130) mmHg, median diastolic blood pressure was 70 (IQR: 60-80) mmHg, median pulse rate was 80 (IQR: 70-91) beats/min, and median respiration rate was 16 (IQR: 15-18) /min in syncope patients at first examination in the ED (Table 2).

QTc's of patients with a history of CAD and with a history of COPD were statistically significantly higher (*p* = 0.009 and *p* = 0.027, respectively) compared to patients without a history of either disease (Table 2). When QTc was evaluated according to gender, the mean value was found to be 425 in men and 428.5 in women, but this relationship was not statistically significant. There was a statistically significant relationship between prolonged QTc interval and mortality (*p* < 0.001).

**Figure 1:**
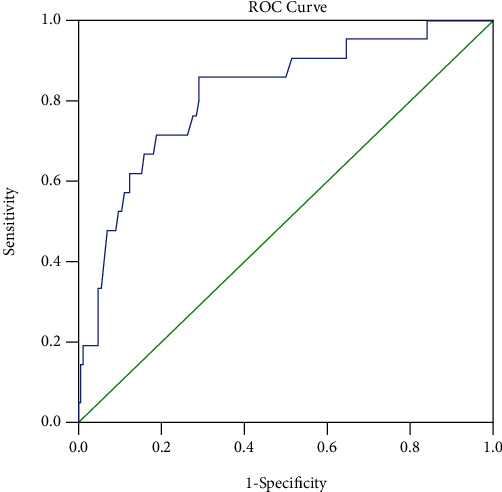
ROC analysis to describe the relationship between mortality and QTc values in syncope patients.

In ROC analysis, to describe the relationship between mortality and QTc values in syncope patients, it was found that a QTc above 440.5 detected mortality with 86% sensitivity and 71% specificity (area under the curve (AUC) = 0.815; 95%CI = 0.71 − 0.91; *p* < 0.001) (Figure 1).

## 4. Discussion

Syncope is a symptom that constitutes 1-3% of admittances to the ED [13]. The QTc interval has been associated with an increased risk of malignant ventricular arrhythmias and with cardiogenic syncope [14]. Spargias et al. suggested that QTc may be caused by a potential underlying cardiovascular disease in syncope patients [15].

Although QTc prolongation can be seen in completely healthy people, it is often accompanied by chronic diseases and may be the cause of ventricular arrhythmia and death in these patients [7, 8, 16]. While studies are showing a significant positive relationship between CAD and prolonged QTc in the literature [17], there are also studies showing the opposite [18, 19]. In this study, QTc interval was found to be statistically significantly higher in patients with a history of CAD compared to those without.

Chronic lung diseases such as COPD are thought to be associated with QTc prolongation, and it is known that QTc prolongation can cause dysrhythmias and increase the risk of mortality in COPD patients [20]. According to another study, the prolongation of QTc interval corrected with Bazett's formula is a common finding in patients admitted with acute respiratory problems. It is a sign of cardiovascular comorbidity in patients with COPD and is associated with increased mortality [21].

In our study, the QTc interval in COPD patients presenting with syncope was found to be statistically significantly higher than in non-COPD patients presenting with syncope. This may be explained by the fact that autonomic neuropathy seen in COPD causes prolongation of ventricular repolarization and QTc prolongation [22].

In some studies, it has been reported that the QTc interval calculated in patients with a history of DM disease is significantly longer than in nondiabetic patients [23, 24]. This is thought to be due to secondary cardiac autonomic neuropathy in diabetic cases [25]. In our study, no statistically significant difference was observed between the QTc interval lengths of diabetic and nondiabetic patients.

In a study by Seftchick et al., it was reported that stroke was a predisposing factor for prolongation in QTc interval [26]. In this study, the length of QTc interval of patients with a history of stroke was not different from those without a history of stroke. This may be due to the low number of patients with a history of stroke in this study.

Hyperthyroidism is associated with QTc prolongation. There are case reports in the literature reporting QTc prolongation in hyperthyroidism, although there is not enough study on this subject [27, 28]. In our study, there was no statistically significant difference between the QTc intervals in patients with and without HT. We found no association of QTc with thyroid disease history. Algra et al. [29] reported that QTc prolongation seen in a patient with hyperthyroidism with impaired thyroid function tests improved after treatment. The fact that all patients with hyperthyroidism were taking antithyroid drugs in our study may explain the normal QTc interval.

In the study conducted by Aggarwal et al., it was reported that a QTC interval above 500 ms indicated increased mortality [17]. Nelson and Leung in their study reported that the risk of sudden cardiac death is three times higher in elderly patients with QTc above 420 ms [7]. In the study of Algra et al. with syncope patients, cases with QTc > 440 ms were associated with increased mortality [29]. In the present study, QTc was found to be higher in patients with mortality compared to survivors. In the Cox regression analysis, it was found that the QTc interval above 440 ms predicted mortality independently.

Often, women have longer QTc than men [30, 31]. In this study, there was no statistically significant difference between the QTc interval calculated in men and women (*p* = 0.867). This may be due to the effect of existing comorbid diseases on QTc interval [30].

## 5. Limitations

The study has some limitations. The small number of patients in the study is the first limitation. Since we excluded the patients who have unpredicted QT prolongation, we could not evaluate all patients presenting with long QT. Multicenter studies with more patients are needed. Another limitation is that only Bazett's formula was used in QTc calculation in this study. There are several alternative formulas available, each with different advantages and disadvantages. Studies including other formulas could be done.

## 6. Conclusion

In this study, we found that the calculation of QTc in patients presenting with syncope can identify the elevated risk of mortality. Qtc prolongation may be a warning parameter for the physician regarding cardiac risk in patients presenting with syncope. It may guide physicians in performing further examinations in these patients. For this reason, QTc prolongation should be evaluated more carefully in patients with syncope, and it should be kept in mind that this may be a marker for underlying cardiac pathologies.

## Figures and Tables

**Table 1:** Demographic data, vital signs, and QTc intervals of cases.

	Mortality	
	Survive	Nonsurvive
Gender		
Female, *n* (%)	60 (88.2)	8 (11.8)
Male, *n* (%)	83 (85.6)	14 (14.4)
CAD history		
Positive, *n* (%)	44 (93.6)	3 (6.4)
Negative, *n* (%)	99 (83.9)	19 (16.1)
HT history		
Positive, *n* (%)	71 (88.8)	9 (11.2)
Negative, *n* (%)	72 (84.7)	13 (15.3)
DM history		
Positive, *n* (%)	35 (87.5)	5 (12.5)
Negative, *n* (%)	108 (86.4)	17 (13.6)
Stroke history		
Positive, *n* (%)	8 (72.7)	3 (27.3)
Negative, *n* (%)	135 (87.7)	19 (12.3)
COPD history		
Positive, *n* (%)	17 (81.0)	4 (19.0)
Negative, *n* (%)	126 (87.5)	18 (12.5)
Thyroid disease history		
Positive, *n* (%)	5 (86.7)	1 (16.7)
Negative, *n* (%)	138 (86.8)	21 (13.2)
Age (year), median (IQR)	64 (79-49)	76 (90-65)
Systolic blood pressure (mmHg) median (IQR)	120 (134-107)	108 (122-98)
Diastolic blood pressure (mmHg) median (IQR)	70 (130-104)	58 (126-102)
Pulse rate (/min) median (IQR)	78 (88-70)	92 (91-70)
Respiratory rate (/min) median (IQR)	16 (19-14)	16 (18-14)

IQR: interquartile range; CAD: coronary artery disease; HT: hypertension; DM: diabetes mellitus; COPD: chronic obstructive pulmonary disease.

**Table 2:** Relationship between QTc and gender, chronic diseases, and mortality.

	Positive		Negative		*p*
	*n*	Median (IQR)	*n*	Median (IQR)	
CAD	47	442 (417-462)	118	422 (398-447)	**0.009**
HT	80	433 (408.5-461.5)	85	422 (398-447)	0.061
DM	40	430 (406.5-459.5)	125	425 (398-454)	0.390
Stroke	11	442 (401-465.5)	154	425 (402-454)	0.620
COPD	21	440 (410-469)	144	424.5 (399-454)	**0.027**
Thyroid disease	6	465 (445-475)	59	425 (401.5-454)	0.282
Mortality	21	470 (444-485)	144	422.5 (398.5-446.5)	**<0.001**

## Data Availability

The data used to support the findings of this study are available from the corresponding author upon request.
